# An Affordable In-house Tubular Retractor for Evacuation of Intracerebral Hematomas: A Case Series and Literature Review

**DOI:** 10.1055/a-2713-5817

**Published:** 2025-10-15

**Authors:** Wilairat K. Kaewborisutsakul, Anukoon Kaewborisutsakul, Surapong Chatpun, Kwunchit Oungbho, Waritorn Srakhao, Kanisorn Sungkaro, Chin Taweesomboonyat

**Affiliations:** 1 Anatomy Program, Faculty of Sciences, Prince of Songkla University, Songkla, Thailand; 2 Department of Surgery, Faculty of Medicine, Neurological Surgery Unit, Prince of Songkla University, Hat Yai, Songkhla, Thailand; 3 Department of Biomedical Sciences and Biomedical Engineering, Faculty of Medicine, Prince of Songkla University, Hat Yai, Songkhla, Thailand; 4 Department of Pharmaceutical Technology, Faculty of Pharmaceutical Sciences, Prince of Songkla University, Hat Yai, Songkhla, Thailand; 5 Translational Medicine Research center, Faculty of Medicine, Prince of Songkla University, Hat Yai, Songkhla, Thailand

**Keywords:** stroke, tubular retractor, endoport, minimal invasive surgery

## Abstract

**Background:**

Intracerebral hemorrhage (ICH) disproportionately affects low- and middle-income countries (LMICs), where prevalence and outcomes are poor. Surgical intervention is often necessary in life-threatening cases. This study explored the feasibility of using a low-cost, in-house tubular retractor for ICH evacuation in a resource-limited setting.

**Methods:**

We retrospectively reviewed adults with spontaneous supratentorial ICH who underwent evacuation with an International Organization for Standardization (ISO)-compliant, in-house tubular retractor (production cost approximately $60) between January 2023 and June 2024. Outcomes included hematoma volume reduction, correction of midline shift, perioperative complications, reoperation, hospital stay, and Glasgow Coma Scale (GCS) scores at discharge and 6 months.

**Results:**

A total of 18 patients (13 males, 5 females; mean age 60.6 ± 13.8 years) underwent surgery. Median hematoma volume was 65.3 cm ^3^ (IQR, 48.5–93.8), with a mean reduction of 81.2% ± 11.7 (median 83.9% [IQR 73.4–88.3]). Midline shift correction averaged 58.5% ± 28.0 (median 55.9% [IQR 43.7–69.6]). Hematoma evacuation was similar whether surgery occurred within 6 hours or later (79.8% vs. 83.5%, *p* = 0.49). Putaminal and frontal hematomas ( *n* = 14) showed greater reduction than non-putaminal ( *n* = 4), though not statistically significant. Median hospital stay was 23.5 days (IQR, 14.5–50.5). At 6 months, median GCS improved from 13 (IQR, 9–14) at discharge to 15 (IQR, 12–15). Two patients died of non-neurological causes.

**Conclusion:**

Use of an in-house, ISO-compliant tubular retractor is feasible and cost-effective for intracerebral hematoma evacuation in resource-limited settings. These preliminary findings support further investigation to refine the technique and assess its clinical impact.

## Introduction

The global impact of intracerebral hemorrhage (ICH) is greatest in low- and middle-income countries (LMICs), where prevalence is highest and outcomes most severe. The incidence of hemorrhagic stroke is markedly higher in LMICs than in high-income countries. Notably, ICH cases in LMICs have risen significantly over the past two decades compared with more affluent regions. 1 Furthermore, ICH in LMICs is associated with high disability and mortality rates, leading to long-term impairments in many survivors. These statistics underscore the urgency for effective health strategies tailored to the unique challenges of limited-resource settings.

Surgical intervention is the standard treatment for life-threatening ICH, particularly when hemorrhage is severe and associated with a low Glasgow Coma Scale (GCS) score at presentation. 2 3 4 5 However, the surgical paradigm for supratentorial ICH has shifted toward minimally invasive approaches that potentially improve functional outcomes. Advancements in endoscopic clot evacuation, keyhole approaches, and tubular access methods have reduced tissue injury, complications, recovery time, and mortality.

The tubular retractor, also referred to as the cylindrical retractor, port system, or parafascicular portal approach, is a vital tool designed based on the keyhole concept of neurosurgery. 6 7 8 9 10 11 This tool enables surgeons to remove a clot under room lighting or with the aid of loupes or a microscope. Despite their utility, commercial tubular retractors are often expensive, making them inaccessible to many hospitals. Consequently, some neurosurgeons have adapted syringes by cutting the distal end to serve as makeshift tubular retractors. 12 13 14 15

However, the use of syringes as tubular retractors has some inherent clinical limitations. Our institutional experience, consistent with previous reports, confirms that although cost-effective, this temporary tool may cause cortical surface trauma, an outcome that is far from ideal. Moreover, using non-standardized equipment in neurosurgery raises legitimate concerns about patient safety due to the lack of critical biocompatibility testing and compliance with medical device standards. These concerns led to the development of an in-house tubular retractor, designed to minimize cortical damage, comply with medical device regulations, and remain economically viable.

**Fig. 1:**
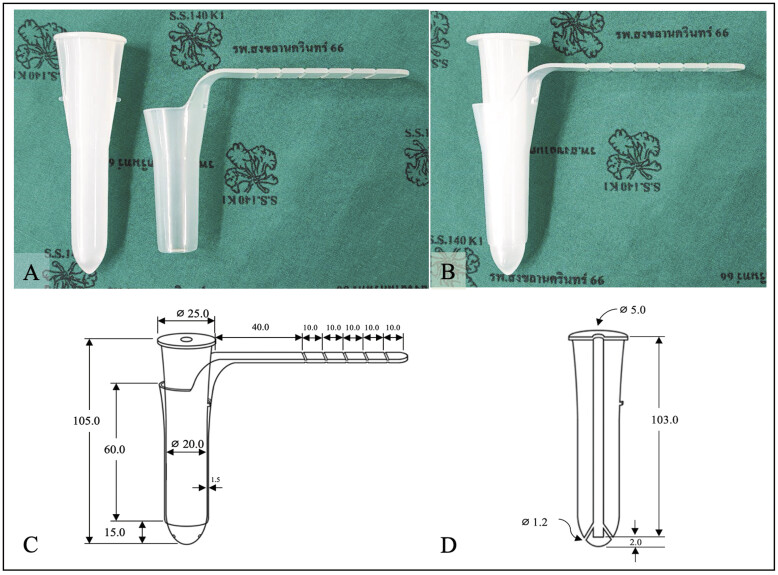
In-house ISO-compliant tubular retractor for minimally invasive neurosurgery. ( **A** ) External tube with extension arm and inner obturator, designed for smooth transcortical navigation. ( **B** ) Fully assembled retractor, prepared for clinical use. ( **C** ) Technical schematic of the assembled tube, showing dimensions (overall length 105 mm, internal diameter 20 mm, wall thickness 1.5 mm, extension arm with depth markings). ( **D** ) Cross-sectional schematic of the obturator, illustrating the blunt-tipped design with central and lateral holes. All dimensions in mm. Symbol ⌀ indicates diameter.

In this study, a tubular retractor was conceptualized and developed in-house using three-dimensional (3D) drawing software to refine the prototype. Production was outsourced to a manufacturer certified in Good Manufacturing Practice (GMP) and International Organization for Standardization (ISO 13485) standards for medical devices, using medical-grade polypropylene and plastic injection molding. The final design comprised an external tube with an extension arm and a blunt-tipped, detachable obturator ( Fig. 1A, B ). The tube measured 105 mm in length, with an internal diameter of 20 mm, an outer diameter of 23 mm (wall thickness, 1.5 mm), and a 40-mm extension arm marked at 10-mm intervals ( Fig. 1C, D ). The retractor was compatible with direct visualization, surgical loupes, and an operating microscope.

Before clinical use, the device underwent ISO 10993 biological safety testing, including cytotoxicity assessment, and passed validated ethylene oxide sterilization by an accredited international laboratory. These measures ensured biocompatibility, sterility, and regulatory compliance for patient use.

We aimed to explore the feasibility of an in-house tubular retractor as an alternative tool for intracerebral hematoma evacuation in resource-limited settings. The primary objective was to demonstrate its potential utility in clot removal for patients with spontaneous supratentorial ICH.

## Materials and Methods

### Study Design and Patient Selection

This pilot retrospective study was conducted at our institution between January 2023 and June 2024 to evaluate the efficacy and safety of an in-house tubular retractor in consecutive patients who underwent craniotomy for hematoma removal. Patients aged >18 years with spontaneous supratentorial ICH were included, while those with hemorrhage secondary to tumors, aneurysms, or arteriovenous malformations were excluded.

### Surgical Procedures

**Fig. 2:**
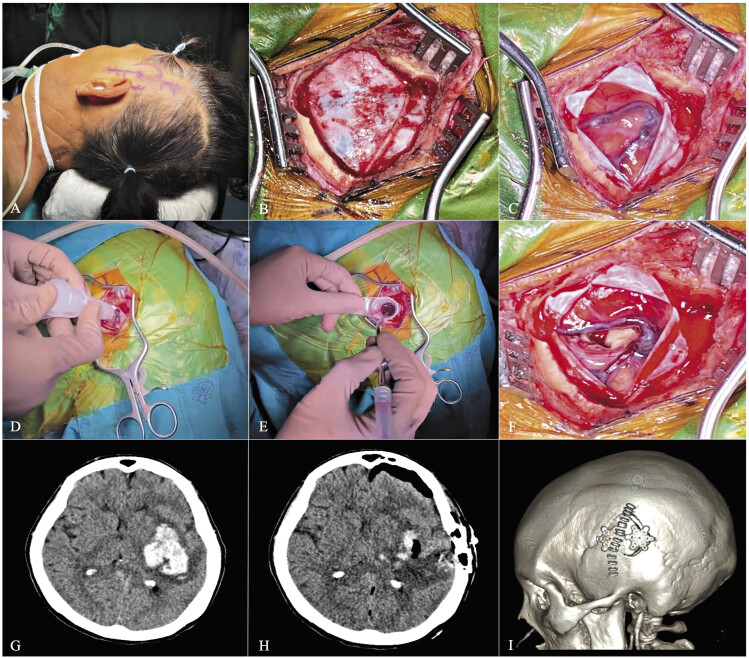
Operative sequences and case demonstration of minimally invasive hematoma evacuation. ( **A** ) Patient No. 2's preoperative preparation, emphasizing the positioning and delineated incision strategy. ( **B** ) The taut dura mater after a 3.5-cm craniotomy. ( **C** ) The cerebral cortex with edema and significant cortical venous presence. ( **D** ) The application of the tubular retractor through a transcortical route guided by intraoperative ultrasonography. ( **E** ) The operative field post inner obturator extraction, with hematoma visualized and removed through suction and irrigation. ( **F** ) The post-evacuation cortical surface showing decreased edema and conserved cortical vessels. ( **G, H** ) Pre- and postoperative CT scans showing the clot at the left putamen and the extensive hematoma evacuation. ( **I** ) A postoperative three-dimensional skull reconstruction marks the incision trajectory and the secured bone flap with staples, plates, and screws.

An expedited surgical procedure was initiated after obtaining informed consent from patients' relatives. General anesthesia was administered at the anesthesiologist's discretion. A senior neurosurgeon (A.K.) performed all procedures with patients positioned on a horseshoe headrest. The craniotomy site was determined by correlating surface anatomy with preoperative computed tomography (CT) imaging, aiming for a 3- to 4-cm diameter bone flap over the cortical site closest to and overlaying the hematoma. A linear or slightly curvilinear incision was made over the midsection of the cranium, followed by a cruciate dura opening upon completion of bone work. The trajectory of the tubular retractor was guided by intraoperative ultrasonography (IOUS), using a transcortical or transsulcal approach to access the hematoma site. The retractor facilitated hematoma removal, primarily through suction and saline irrigation, under visual enhancement of an operating microscope when necessary. After removal, the surgical field was reassessed with IOUS to confirm hematoma clearance, and dural closure was achieved using watertight techniques. The bone flap was secured with plates and screws. Notably, all patients were monitored in the intensive care unit. A routine CT scan was performed within 12 h postoperatively to evaluate any residual hematoma and to check for surgical complications ( Fig. 2 ). Postoperative care included early enteral nutrition, catheter removal, and early rehabilitation, with discharge upon satisfactory recovery and readiness for home care.

### Outcome Measurement

The primary outcome was the feasibility of the in-house tubular retractor, assessed by its ability to access the hematoma, perform evacuation, and achieve measurable volume reduction and midline shift correction. Secondary outcomes included perioperative data (complications, reoperation rates, length of stay, and GCS scores at discharge and 6-month follow-up) recorded for observational analysis.

### Statistical Analysis

Data were collected from the hospital database with institutional ethics approval (REC.66-262-10-1). Descriptive statistics were used, with results expressed as numbers, percentages, means ± standard deviations, medians, and ranges, as appropriate for data type and distribution.

## Results

This study included 18 patients (13 males, 5 females) with a mean age of 60.6 ± 13.8 years (range 24–81). Hypertension was the most frequent comorbidity. Hematomas were evenly distributed between hemispheres, predominantly in the putamen (13 patients), followed by the thalamus (2 patients), frontal lobe (1 patient), and parietal or parieto-temporal regions (2 patients). Preoperative GCS had a median of 7.5 (IQR 6.3–9.0), ranging from 3 to 12, reflecting variability in initial severity. All patients required intubation on arrival at the emergency department. Median hematoma volume was 65.3 cm ^3^ (IQR 48.5–93.8) (range 17.0–209.3), and mean midline shift was 10.2 ± 5.4 mm (range 1.5–21.0). Median ICH score was 3 (IQR 2–3) (range 2–5) ( Table 1 ).

**Table 1:** Characteristics of patients who underwent tubular retractor-assisted intracerebral hemorrhage removal ( *n* = 18)

Patient no.	Age (year)	Sex	Comorbidity	Antiplatelet/ Anticoagulant	Location, side	IVH	Preoperative GCS	ICH volume (cm ^3^ )	Midline shift (mm)	ICH score
1	68	F	HT, DM	−	Putamen, Rt	Yes	7	62.7	13.0	3
2	69	M	HT	−	Putamen, Lt	−	8	32.4	1.5	2
3	24	M	HT	−	Thalamus, Rt	Yes	7	33.2	5.3	3
4	65	M	−	−	Putamen, Rt	−	9	88.8	11.1	2
5	59	M	HT	−	Putamen, Lt	Yes	3	209.3	21.0	4
6	47	M	−	−	Putamen, Lt	−	9	48.6	4.6	2
7	49	M	−	−	Putamen, Lt	−	9	52.0	7.0	2
8	81	F	HT	ASA	Putamen, Lt	Yes	7	93.0	17.7	4
9	61	M	−	−	Frontal, Rt	−	11	41.7	3.0	2
10	63	M	HT	−	Putamen, Rt	−	6	115.2	10.5	2
11	61	M	HT	−	Putamen, Rt	Yes	5	112.7	15.3	3
12	51	M	Cirrhosis	−	Putamen, Lt	Yes	12	109.7	7.4	3
13	56	M	−	−	Putamen, Rt	Yes	6	94.1	7.6	3
14	71	M	Hypothyroidism	ASA	Thalamus, Lt	Yes	7	17.0	14.8	2
15	81	F	Previous stroke, HT	ASA	Putamen, Lt	Yes	4	53.1	17.3	5
16	53	M	Valvular heart disease	Heparin	Parietal, Lt	Yes	9	67.8	9.1	3
17	77	F	−	−	Parieto-temporal, Lt	−	10	91.5	8.3	2
18	54	M	HT	−	Putamen, Rt	Yes	11	48.4	9.5	3

Abbreviations: ASA, aspirin; DM, diabetes mellitus; F, female; GCS, Glasgow Coma Scale score; HT, chronic hypertension; ICH, intracerebral hemorrhage; IVH, intraventricular hemorrhage; Lt, left; M, male; Rt, right.

The interval from onset to anesthetic induction had a median of 4.6 h (IQR 3.5–8.5) (range 2.5–23). Patients referred from external facilities had longer preoperative intervals. Mean surgical duration was 154.4 ± 71.2 min (median 132.5 [IQR 110–207.5]; range 40–290). Postoperatively, residual hematoma volume had a median of 9.9 cm ^3^ (IQR 5.2–17.0), corresponding to a mean reduction of 81.2 ± 11.7% (median 83.9% [IQR 73.4–88.3]; range 53.2–97.5%). Patients operated within 6 h of onset ( *n* = 11) had a mean reduction of 79.8 ± 13.5%, compared with 83.5 ± 8.8% in later cases ( *n* = 7), with no significant difference ( *p* = 0.49). Midline shift reduction averaged 58.5 ± 28.0% (median 55.9% [IQR 43.7–69.6]; range 11–100%), reinforcing the technique's effectiveness in reducing mass effect ( Table 2 ).

**Table 2:** Operative profiles and postoperative outcomes of patients who underwent the tubular retractor-assisted intracerebral hemorrhage removal ( *n* = 18)

Patient no.	Onset time to surgery (h)	Operative time (min)	Postoperative hematoma volume (cm ^3^ )	Hematoma volume reduction (%)	Midline shift reduction (%)	Perioperative complications	Hospital stays (days)	Postoperative GCS	
								At discharge	At 6 months follow-up
1	14.1	140	9.1	85.5	59.9	−	13	15	15
2	10	120	5.2	84.0	100	−	20	11	15
3	3.2	110	5.7	82.8	100	Hydrocephalus	19	13	15
4	23	60	10.2	88.5	64.2	Pneumonia	52	15	15
5	2.8	110	98.0	53.2	21.9	Prolong intubation, hydrocephalus	46	5	8
6	4.7	150	3.9	91.9	100	−	9	13	15
7	6.3	200	2.1	96.0	100	−	9	14	15
8	3.6	85	29.3	68.5	51.4	Prolong intubation	12	9	11
9	5.7	125	2.2	94.7	66.7	−	8	15	15
10	4.5	100	31.2	72.9	57.1	Prolong intubation, hydrocephalus	19	9	9
11	9.5	40	28.2	75.0	70.6	Prolong intubation, hydrocephalus	30	8	12
12	2.5	120	13.5	87.7	44.6	Pneumonia	36	14	15
13	3.0	240	15.2	83.8	28.9	Prolong intubation	20	9	12
14	9.2	190	5.2	69.4	54.1	Prolong intubation, pneumonia, UTI	84	11	15
15	3.5	210	17.6	66.9	11.0	Prolong intubation, UTI	97	Death (palliative care after postoperative)	
16	6.1	230	9.6	85.8	24.2	Prolong intubation, heart failure	80	10	Death (due to cardiac condition)
17	4.5	290	2.3	97.5	43.4	UTI, epilepsy	70	12	12
18	4.0	260	10.8	77.7	54.7	Lung atelectasis	27	14	15

Abbreviations: GCS, Glasgow Coma Scale score; UTI, urinary tract infection.

In subgroup analysis, putaminal and frontal hematomas ( *n* = 14) showed higher median volume reduction (83.8% vs. 76.1%) and greater midline shift correction (57.1% vs. 49.4%) than non-putaminal locations ( *n* = 4), though differences were not significant ( *p* = 0.52 and *p* = 0.09, respectively).

Perioperative complications included postoperative hydrocephalus requiring ventriculoperitoneal shunt in four of nine patients (44.4%) with intraventricular hemorrhage (IVH). Prolonged intubation was documented in eight patients, with tracheostomy required in several cases. Other complications included pneumonia (three), urinary tract infection (three patients), epilepsy (one patient), lung atelectasis (one patient), and heart failure (one patient). Median hospital stay was 23.5 days (IQR 14.5–50.5), with a mean of 36.2 ± 28.7 days (range 8–97). At follow-up, two patients had died (one after postoperative decline under palliative care, one from cardiac disease), while the remainder were discharged with scheduled outpatient follow-up ( Table 2 ).

Recovery outcomes, assessed with GCS, showed a median score of 13 (IQR 9–14) at discharge (range 5–15). Median GCS improved to 15 (IQR 12–15) at 6 months (range 8–15). Three patients did not regain a GCS of 15 at 6 months. Two patients (Nos. 5 and 8) had initial ICH scores of 4 and showed limited recovery. Conversely, one patient (No. 10) with an initial ICH score of 2 did not achieve a favorable outcome despite a seemingly better baseline. This discrepancy may be explained by larger hematoma size, lower volume reduction, reduced midline shift correction, and complications, including prolonged intubation and postoperative hydrocephalus.

## Discussion

Hemorrhagic stroke is a serious health concern globally, particularly in LMICs, where it is associated with higher mortality rates compared to high-income nations. 1 5 Traditional surgical interventions are crucial in preserving life, particularly in cases of extensive superficial ICH without IVH; however, their effectiveness in enhancing postoperative functional outcomes remains uncertain, as evidenced in the Surgical Treatment for Ischemic Heart Failure trial. 16 This uncertainty is compounded by unresolved questions regarding the benefits of surgery, optimal timing of surgical intervention, and selection of the most appropriate surgical approach. Consequently, these knowledge gaps necessitate further investigation to inform clinical decision-making and optimize patient care.

The present study assessed the effectiveness of an in-house–developed tubular retractor for ICH removal in a limited resource setting. A total of 18 patients, most with putaminal hematomas, underwent the procedure. Mean hematoma volume reduction was 81.2 ± 11.7% (median 83.9% [IQR 73.4–88.3]), and mean midline shift correction was 58.5 ± 28.0% (median 55.9% [IQR 43.7–69.6]). Despite complications such as hydrocephalus, pneumonia, and prolonged intubation with tracheostomy, median GCS improved from 13 (IQR 9–14) at discharge to 15 (IQR 12–15) at 6 months. These findings indicate the potential of an in-house tubular retractor as a safe and effective tool for managing ICH in LMICs.

Minimally invasive surgical (MIS) techniques, such as the use of tubular retractors, are promising. These retractors, which are pivotal in MIS, can lead to enhanced patient outcomes by allowing precise targeting of hematomas, reducing brain tissue trauma and facilitating recovery. 17 18 19 These benefits are based on their ability to enable near-total hematoma removal with minimal complications. Notably, the 2022 American Heart Association guidelines for managing spontaneous ICH highlight that MIS may be a viable option for enhancing functional outcomes compared with conventional craniotomy techniques. 2 However, despite these advances offering substantial improvements in surgical visualization and patient recovery times, the cost of these innovative tools often hinders their widespread use in LMICs.

The timing of surgical intervention further complicates its clinical application. There is an ongoing debate regarding the benefits of early surgery, which states that early intervention may be associated with poorer outcomes owing to clot instability. This controversy is reflected in the varying results reported in comparative studies. 20 21 22 23 However, a recent randomized trial has provided further insights. The Early Minimally Invasive Removal of Intracerebral Hemorrhage (ENRICH) trial, which evaluated the efficacy of MIS using the BrainPath minimal access port and Myriad device (NICO Corporation, Indianapolis, IN, USA) for ICH within 24 h of symptom onset, demonstrated that early surgical intervention using the tubular approach significantly improved functional outcomes at 180 days compared to standard medical management. Specifically, patients who underwent minimally invasive hematoma evacuation had a mean utility-weighted modified Rankin Scale (uW-mRS) score of 0.458 compared to the 0.374 score obtained in the medical management group, with a posterior probability of superiority of 0.981, surpassing the predefined threshold for superiority. 24

Notably, various factors influence the outcome of patients with ICH, underscoring the complex nature of this condition. A pivotal factor is the ICH score, which has been consistently correlated with 30-day mortality rates. 25 Our dataset corroborates this finding, revealing that patients with an ICH score of 4 had poor GCS scores at the 6-month follow-up. This correlation emphasizes the predictive value of the ICH score for long-term patient prognosis. In addition, the residual volume of a postoperative hematoma critically determines patient survival and independence within 1 year after MIS ICH removal. 26 This relationship is attributed to the symptomatic mass effect and secondary injury caused by an unevacuated hematoma. Therefore, meticulous surgical techniques aimed at maximizing hematoma evacuation are required to improve patient outcomes after MIS for ICH removal.

**Fig. 3:**
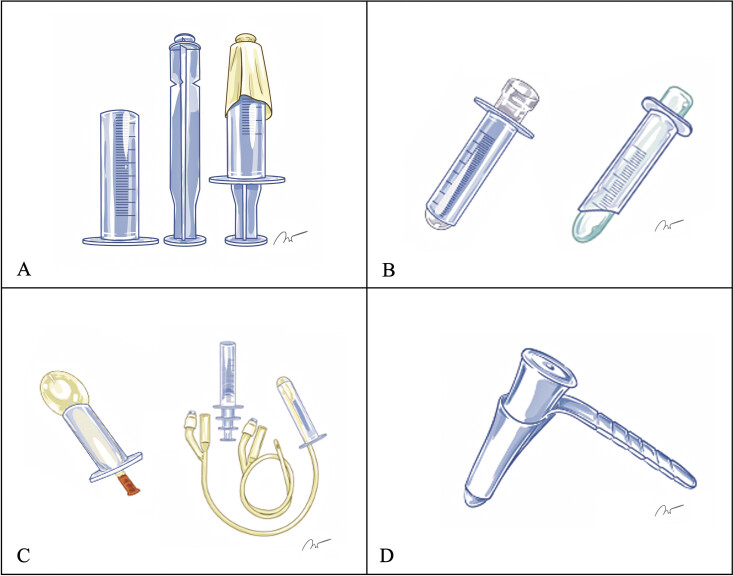
The current designs of the in-house–developed improvised syrinx port system. ( **A** ) Improvised syrinx-based tubular retractor. ( **B** ) Modified inner obturator designs incorporating a centrifuge tube (left) and custom-molded silicone (right). ( **C** ) Obturator designs with balloon fixation using a finger glove (left) and a urinary catheter balloon (right). ( **D** ) Medical-grade material model employed in this study. Medical Illustrations by Vistawash Tang-on.

The design of tubular retractors originally included an outer sheath coupled with an inner obturator or trocar to form the core functional components. 6 These designs also help create a small corridor to access deep-seated lesions and reduce surrounding brain tissue injury compared with conventional spatula retractors. 27 28 This design standard is part of a broader evolution within LMICs, from improvised modifications of medical syringes to more sophisticated engineered solutions. 29 The earlier local adaptations, although ingenious, were limited by practical constraints and potential safety concerns. Transitioning from simple plunger-based models to those using centrifuge tubes, 30 custom-made silicone, 31 finger glove balloon devices, 32 or urinary catheter balloons, 33 some institutions have advanced 3D printing with medical-grade resin for immediate surgical use ( Fig. 3 ). 34

Our in-house tubular retractor was meticulously developed to circumvent the complications observed in previous adaptations in LMICs, where syringes were repurposed as retractors. Our experience with these improvised syringes revealed a risk of cortical injury due to the sharp edges and constraints due to the limited size of available syringes, which were mostly 5-mL or 10-mL syringes. 12 29 30 31 32 33 Furthermore, both improvised syringe port systems and 3D-printed retractors lack standard medical device testing. 34 ISO 10993 outlines a comprehensive assessment of the safety of medical devices, particularly biocompatibility testing. 35 36 This test is fundamental for medical device development and regulatory approval processes, ensuring their safety and compatibility when in contact with biological tissues such as the brain parenchyma. This led to the design of our model, which followed the ISO standard and had a low price of approximately 60 USD per unit. We broadened our review to include improvised, 3D-printed, commercial, and in-house tubular retractors. Improvised syringe- or glove-based devices (<5 USD) are extremely inexpensive but pose risks of cortical injury, are size-limited, and lack ISO certification or sterility validation. 3D-printed retractors (5–50 USD) are customizable and affordable but are rarely manufactured under medical device regulations or validated for safety and reproducibility. Commercial systems such as BrainPath or VBAS are ISO-certified, well-validated, and safe, but prohibitively expensive (>1,500 USD) and largely inaccessible in LMICs. By contrast, our in-house retractor (approximately 60 USD) is moderately priced, biocompatibility-tested, and ISO/GMP-compliant, offering a balanced and sustainable option for LMIC settings ( Table 3 ). To date, adapted or in-house designs have only been reported in case series, with Garcia-Estrada et al demonstrating the benefits of 3D-printed retractors over open surgery. 34 However, no direct comparative study of in-house versus commercial retractors has been conducted.

**Table 3:** Comparison of tubular retractor alternatives for intracerebral hematoma evacuation

Type of retractor	Cost (approximately per unit) a	Safety/Biocompatibility tested	Practicality in LMICs	Key limitations
**Improvised syringe** 30 31 32 33	**Low** <5 USD	**No** No ISO testing; sharp edges with risk of cortical trauma	**High** Widely available; very low cost; easily improvised	Non-standardized; not validated for sterility; unstable corridor
**3D-printed retractors** 34	**Low to medium** 5–50 USD	**No** Usually not biocompatibility-tested after printing; sterility validation rarely performed	**Medium to high** Customizable; rapid prototyping possible	Lack of regulatory approval; variable durability and strength
**Commercial retractors (e.g., BrainPath, VBAS)** 7 8	**High** >1,500 USD	**Yes** ISO-certified; biocompatibility tested	**Low** Clinically validated; available in HICs; single-use in many systems	Cost-prohibitive in LMICs; limited availability
**In-house ISO-compliant tubular retractor (this study)**	**Medium** ∼60 USD	**Yes** ISO-tested (cytotoxicity and sterility PASS); medical-grade polypropylene; GMP/ISO 13485 manufacturing	**Medium to high** Affordable; reusable after sterilization; compatible with microscope/endoscope	Limited to single size in current design; preliminary clinical data only

Abbreviations: GMP, Good Manufacturing Practice; HICs, high-income countries; ISO, International Organization for Standardization; LMICs, low- and middle-income countries.

a Costs are approximate and may vary by region and manufacturer.

Notably, some centers have integrated tubular retractors with endoscopic clot removal as a less invasive approach; however, the endoscopic equipment requires specialized personnel for preparation. This information is often unavailable during emergencies, which is a common scenario in LMICs. Thus, microscopic techniques are available. Furthermore, they have been reported to yield better outcomes in some studies. 37 The choice of visualization tools, whether loupes or microscopes, depends on the depth and consistency of the hematoma and the diameter of the retractor. Sharif et al suggested that syringe-based systems are more compatible with microscopic assistance and provided a tailored solution in settings with limited access to advanced equipment. 13

### Study Limitations

This study had some limitations. First, it was preliminary and based on a small cohort from a single institution, which may have limited the generalizability of its findings. Second, the uniformity of the tubular retractor size used in the procedures could have influenced the outcomes because only one dimension was employed, potentially limiting its applicability across varying hematoma locations and volumes. Third, this study focused on supratentorial ICH, predominantly within the putamen and thalamus, which presents a selection bias and overlooks the diversity in hemorrhagic stroke locations. Fourth, a comparative study between the in-house retractor and commercial models was not conducted. Fifth, the parafascicular approach, which shows evidence of reduced neural pathway damage and postoperative neurological deficits, was not considered. 38 This approach requires tractography imaging, which may be unavailable in most LMIC hospitals, particularly in emergency settings. Lastly, although functional outcome measures such as the modified Rankin Scale (mRS) are more widely recommended in ICH studies, they were not routinely documented in our retrospective dataset. Therefore, we used the GCS, which was consistently available.

### Future Directions

The pursuit of enhanced surgical management of ICH in LMICs requires a multi-dimensional research approach. Therefore, to prioritize patient safety, future endeavors must extend beyond immediate surgical outcomes to include comprehensive evaluations of in-house tool development and to ensure adherence to international standards of biocompatibility. Comparative studies are essential for validating the efficacy and cost-effectiveness of local innovations versus established equipment and techniques, with an emphasis on advocating public policies that support the dissemination of these advancements. Therefore, by aligning research with socioeconomic contexts and health system capabilities, surgical interventions can be tailored to meet the unique needs of LMIC populations, ultimately improving ICH management and patient care. Future prospective studies should also incorporate functional outcome measures, such as the mRS, to better capture long-term neurological recovery.

## Conclusion

This study demonstrates the efficacy and safety of an in-house–developed tubular retractor for the minimally invasive evacuation of intracerebral hematomas. Our findings indicate a significant reduction in hematoma volume and correction of midline deviation, highlighting the potential of the retractor to enhance surgical outcomes in limited-resource settings. However, despite perioperative complications being comparable to those reported in the literature, the overall positive recovery trajectories, as evidenced by the improved GCS scores, support the broader adoption of this innovative tool in neurosurgical practice. Future studies should investigate its applicability across diverse clinical scenarios to validate its effectiveness and safety in various clinical settings.
